# Prediction of Pathologic Complete Response in Esophageal Squamous Cell Carcinoma Using Preoperative Serum Small Ribonucleic Acid Obtained After Neoadjuvant Chemoradiotherapy

**DOI:** 10.1245/s10434-024-16247-z

**Published:** 2024-10-17

**Authors:** Ryosuke Hirohata, Yuki Yamamoto, Takahiro Mimae, Yoichi Hamai, Yuta Ibuki, Ryou-u Takahashi, Morihito Okada, Hidetoshi Tahara

**Affiliations:** 1 Department of Surgical Oncology, Hiroshima University, Hiroshima, Japan; 2 Department of Cellular and Molecular Biology, Graduate School of Biomedical and Health Science, Hiroshima University, Hiroshima, Japan

**Keywords:** Esophagus, Neoadjuvant chemoradiation therapy, Pathological complete response, Serum small RNA, Squamous cell carcinoma

## Abstract

**Background:**

The authors hypothesized that small ribonucleic acid (sRNA) obtained from blood samples after neoadjuvant therapy from patients treated with neoadjuvant chemoradiation therapy (NACRT) could serve as a novel biomarker for predicting pathologic complete response (pCR).

**Methods:**

This study included 99 patients treated with esophagectomy after NACRT between March 2010 and October 2021 whose blood samples were collected between the end of NACRT and surgery. Next-generation sequencing (NGS) was used to analyze sRNAs from the blood samples. A predictive model for pCR comprising micro-RNA isoforms (isomiR), transfer RNA (tRNA)-derived sRNAs (tsRNAs), and clinical factors was constructed using cross-validation.

**Results:**

Of the 99 patients, pCR was diagnosed for 30 and non-pCR for 69 of the patients. Among sRNAs, the isomiRs of let-7b and miR-93 and the tsRNA group derived from tRNA-Gly-CCC/GCC were identified as predictive factors. The clinical factors included a decrease in the maximum standardized uptake value (SUVmax) at the primary site, clinical complete response post-NACRT, preoperative biopsy, and post-NACRT carcinoembryonic antigen levels. The combined predictive model for pCR (C-PM) was established using the three sRNAs and four clinical factors. The area under the curve for the C-PM was 0.84, which was a significant factor in the multivariate analysis (odds ratio, 89.41; 95 % confidence interval 8.1–987.5; *p* < 0.001).

**Conclusions:**

Pathologic complete response after NACRT can be predicted by a predictive model constructed from preoperative clinical factors obtained via minimally invasive procedures and sRNA identified by NGS. Preoperative pCR prediction may influence treatment decision-making after NACRT.

**Supplementary Information:**

The online version contains supplementary material available at 10.1245/s10434-024-16247-z.

Neoadjuvant chemoradiotherapy (NACRT) followed by surgery is the generally accepted standard therapy for resectable advanced esophageal squamous cell carcinoma (ESCC)^1–3^ Complete disappearance of the primary tumor after esophagectomy following NACRT and disappearance of metastatic lymph nodes after neoadjuvant therapy (NAT) indicate a pathologic complete response (pCR).

The pCR rate after NACRT is relatively high (30–40%).^2,4^ As a surrogate marker, pCR after NAT is strongly associated with patient prognosis.^5–7^ Additionally, the possibility of avoiding highly invasive esophagectomy for patients with a diagnosis of a clinical complete response (cCR) has recently been discussed.^8,9^ The prediction of pCR before surgery supports active surveillance and potentially broadens therapeutic strategies.

Non-coding ribonucleic acids (ncRNAs) are RNAs transcribed from deoxyribonucleic acid (DNA) that do not encode proteins. The ncRNAs can be classified into various categories depending on their length, shape, and location. MicroRNAs (miRNAs) are small RNA (sRNA) molecules comprising approximately 20–25 nucleotides. By suppressing messenger RNAs, miRNAs can suppress or promote tumor growth.^10,11^

With the recent development of next-generation sequencing (NGS), an isoform that differs slightly in sequence from mature miRNAs has been identified as an isoform of miRNA (isomiR). The functions of isomiRs have not been fully elucidated, but they play important roles in cancer development.^12^ Transfer RNA-derived sRNAs (tsRNAs), products from the cleavage of transfer RNA (tRNA), a class of ncRNAs, are associated with transcription and translation, as well as with the regulation of cell growth, apoptosis, metastasis, and immunity.^13,14^

Cancer cells possess various ncRNAs, which are present in the bloodstream as inclusions in exosomes.^15,16^ Comprehensive sequencing, represented by NGS, has been used to search for ncRNAs derived from blood samples.^17–21^ Efforts have been made to predict the efficacy of NAT in esophageal cancer treatment by identifying blood-derived ncRNAs, represented by miRNAs.^22–24^ However, reports on ncRNAs derived from blood samples after NAT are limited.

Therefore, we hypothesized that sRNAs obtained from blood samples after NAT from patients treated with NACRT could be used as a treatment response indicator to predict pCR.

## Methods

### Patients and Clinical Parameters

Between March 2010 and October 2021, the study enrolled 99 patients treated with esophagectomy after NACRT whose blood samples were obtained between the completion of NACRT and surgery. The study of predicting pCR after NACRT by small RNA was approved by The Institutional Review Board of Hiroshima University (Ethical Committee for Epidemiology of Hiroshima University, HI-129).

All the tumors were histologically diagnosed as ESCC based on biopsy samples. The following patient clinical parameters were collected: age, sex, tumor-node-metastasis (TNM) stage, tumor diameter, percentage change in the maximum standardized uptake value (SUVmax) of the primary tumor before and after NACRT (%ΔSUVmax) on positron emission tomography-computed tomography (PET-CT), carcinoembryonic antigen (CEA) levels, squamous cell carcinoma (SCC) antigen levels after NACRT, overall response, endoscopic findings, and biopsy results after NACRT. The TNM classification was based on the eighth edition of the Union for International Cancer Control.^25^ The criteria to determine the overall cCR required that the primary site be judged as CR by endoscopy, that the metastatic lymph node be judged as CR by RECIST,^26^ and that tumor marker levels be within the normal range.

### NACRT, Surgery, and Pathologic Findings

The NACRT comprised concurrent radiotherapy (40 Gy) and chemotherapy with 5-fluorouracil, docetaxel, cisplatin, nedaplatin, or a combination of both. Esophagectomy with at least two-field (thoracic and abdominal) lymph node dissection was scheduled for all the patients 4 to 8 weeks after the completion of NACRT.

The pathologic treatment response of the primary tumor was judged based on the criteria of the Japan Esophageal Society as follows: grade 0 (no recognizable cytologic or histologic therapeutic effect), grade 1 (slightly effective, with apparently visible cancer cells accounting for at least one third of the tumor tissue), grade 2 (moderately effective, with visible cancer cells accounting for less than one third of the tumor tissue), and grade 3 (markedly effective, with no evidence of visible cancer cells). Grade 3 treatment effect after NACRT of the primary tumor and the presence of no metastatic lymph nodes in the resection specimen (pT0N0M0) was defined as pCR. The presence of residual tumor cells in both or one of the primary sites and metastatic lymph nodes, including grade 3 primary tumors and residual lymph node metastases, was defined as non-pCR.

### Library Preparation and sRNA Sequencing Using NGS

Peripheral blood samples were collected from each patient between the end of NACRT and surgery, separated via centrifugation at 3000 rpm for 10 min at 4 °C, and stored at − 80 °C. Total RNA was isolated from 200 µL of serum using the miRNeasy Mini Kit (Qiagen), following the manufacturer’s protocol. The NEXTFLEX sRNA-Seq Kit v3 (PerkinElmer) was used to prepare a reconstructed complementary DNA library from 2 ng of serum-derived RNA, following the manufacturer’s protocol. The amount and integrity of the amplified complementary DNA were assessed using an Agilent 2100 Bioanalyzer (Agilent Technologies) with a high-sensitivity DNA assay chip, following the manufacturer’s protocol. The libraries were sequenced on a NextSeq 2000 (Illumina) with the P2 100 cycle kit for a single-end 50-cycle run, following the manufacturer’s instructions.

### Analysis of Sequencing Results

The obtained sequence data were imported into the CLC Genomics Workbench 7 (CLCbio) and analyzed. For the sequences obtained, mismatches of up to two nucleotides were allowed for identification. The total number of reads was adjusted to 1,000,000, and the resulting values were corrected to the normalized value of the respective library to normalize the measured reads. The normalized reads were annotated using miRBase version 21. Let-7a, miR-223, miR-197, miR-574-3p, miR-150, miR-451, miR-16, miR-92a, miR-486-5p, and miR-122, reported to be associated with hemolysis, were excluded from the original data for analysis.^27^ The remaining reads were annotated using hg19-tRNA. The tsRNAs were treated as a tsRNA group and classified by the source name of the mature tRNA. Fragments derived from the same tRNA reference sequences with a common sequence on the 5′ side were identified using the same name. In the tsRNA analysis, mismatches were corrected in accordance with the original tRNA sequence, and normalized expression values of each identifiable tsRNA were summed and calculated for each blood sample. Candidate sRNAs were identified using the following criteria: detected in more than 80 % of patients with pCR, with median normalized values of pCR and non-pCR exceeding 100.

### Statistical Analysis and Establishment of the pCR Predictive Model Using Cross-Validation

Continuous variables are presented as medians (ranges) and categorical variables as numbers (percentages). Continuous variables were compared using the Mann–Whitney *U* test and categorical variables using the chi-square test. Logistic regression analysis was performed using candidate clinical factors to establish a predictive model from clinical factors. Factors with *p* values lower than 0.1 were selected as explanatory variables. Logistic regression analysis was performed by sequentially combining sRNAs in the order of odds ratios from the cross-validation results to establish a predictive model for sRNAs based on NGS data.

The best diagnostic model was adopted by comparing the pCR prediction ability of each model. All data were cross-validated five times with a learning-to-validation ratio of 8:2 to create a regression model set for optimal hyperparameters. A combination model using all explanatory variables in the predictive model with clinical factors and a predictive model with sRNAs was created and tested for its ability to predict pCR. The analysis used to establish the predictive model was outsourced to Rejoui Inc. (Tokyo, Japan).

Prognoses were investigated in terms of recurrence-free survival (RFS) and overall survival (OS), with RFS defined as the time from the date of surgery to the first event (recurrence or death from any cause) or the most recent follow-up visit and OS defined as the time from the date of surgery to the date of death from any cause or until the most recent follow-up visit.

Cox regression analysis included all covariates in the multivariate analysis. Kaplan–Meier curves were compared using the log-rank test. Statistical significance was set at a *p* value lower than 0.05. Survival outcomes were evaluated in January 2023. All statistical analyses were performed using SPSS version 29.0 (IBM Corp., Armonk, NY, USA).

## Results

### Clinical and Pathologic Background

Of the 99 cases included in the analysis, 30 were classified as pCR and the remaining 69 as non-pCR. Of the 69 patients in the non-pCR group, 11 achieved CR in the primary tumor (grade 3) after NACRT but had residual cancer in the regional lymph nodes (Table S1).

Regarding postoperative recurrence, 1 (3.3 %) patient in the pCR group had local recurrence, and 5 (16.6 %) patients had recurrence with distant metastasis. However, in the non-pCR group, 9 (13.9 %) patients had recurrence with local metastasis, and 31 (44.9 %) patients had recurrence with distant metastasis. Both recurrence patterns more common in the non-pCR group (*p* = 0.002). Among the patients with pCR, 6 (20.0 %) died of esophageal cancer, and 7 (23.3 %) died of other diseases. Among those without pCR, 36 (52.2 %) died of esophageal cancer, and 11 (15.9 %) died of other diseases.

### Predictive Model of pCR With Isomirs and tsRNAs

**Fig. 1:**
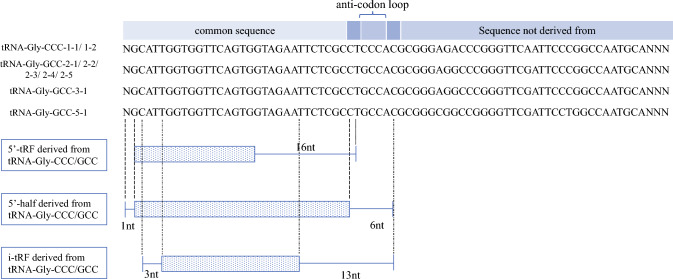
Original nucleotide sequence of the candidate transfer ribonucleic acid (tRNA) Gly-CCC/GCC-derived small RNA (tsRNA group derived from tRNA-Gly-CCC/GCC) and classification by cleavage site. The 5′-transfer RNA is classified as starting from the 5′ side of the original sequence and not reaching the anticodon loop, the 5′-half as reaching the anticodon loop, and the i-transfer RNA as starting in the middle of the original sequence. The dotted bars in the figure indicate the common sequence, and the lined areas indicate the differences in the sequence

The study identified 35 isomiRs and 32 tsRNAs as sRNAs that met the analysis criteria (Supplementary File 1). The diagnostic ability of the pCR predictive model was evaluated by increasing the number of explanatory variables in the order of increasing odds ratios, as determined via cross-validation. One to three candidate sRNAs were combined in the order of increasing odds ratios. The candidate sRNAs included in these three models were let-7b-5p isoforms (3' deletion T and 3′ addition CTC), miR-93-5p isoforms (3'addition TTTG), and a tsRNA group derived from tRNA-Gly-CCC-1-1/1-2, tRNA-Gly-GCC-2-1/2-2/2-3/2-4/2-5/2-6, tRNA-Gly-GCC-3-1, and tRNA-Gly-GCC-5-1 (tsRNA group from tRNA-Gly-CCC/GCC). These tsRNAs derived from tRNA-Gly-CCC/GCC were classified into 5′-tRNA-derived fragments (tRFs), 5′-halves, and internal-tRFs (i-tRFs) based on the cleavage sites of the reference sequence of the original tRNA (Fig. 1).

**Fig. 2:**
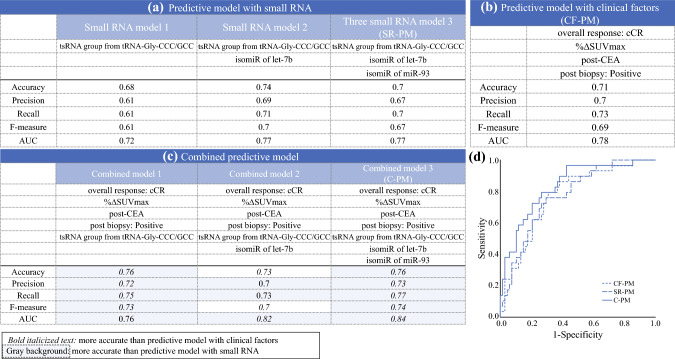
Predictive ability of (**a**) the predictive model with small RNAs, (**b**) the model with clinical factors, and (**c**) the combined predictive model; (**d**) the receiver operating characteristic curve of each predictive model. In each combined predictive model, the superiority over the predictive model with clinical factors is shown in bold font and over that with small RNAs with a gray background

Table 1 shows the comparison of the three candidate sRNAs in the pCR and non-pCR groups. The levels of the Gly-CCC/GCC tsRNAs significantly differed between the pCR and non-pCR groups when they were considered collectively rather than differentiated by 5'-tRF, 5'-half, or i-tRF, indicating that they were included in the same group. Figure 2a shows the accuracy, precision, recall, F-measure, and area under the curve (AUC) of the pCR predictive model with one to three sRNAs. A predictive model including the three candidate sRNAs was adopted as the predictive model with sRNA (SR-PM). The SR-PM was established with the following equation:

$$\begin{aligned} {\text{Score}} = & - {1}.{7445 } + \, 0.00{3717 } \times [{\text{isomiR}}\,{\text{of}}\,{\text{let}} - {\text{7b}} - {\text{5p}}] + \, 0.00{3414 } \\ & \times \,[{\text{tsRNA}}\,{\text{group}}\,{\text{from}}\,{\text{ Gly}} - {\text{CCC}}/{\text{GCC}}] + \, 0.000{343} \\ & \times \,[{\text{isomiR}}\,{\text{of}}\,{\text{miR}} - {93} - {\text{5p}}]. \\ \end{aligned}$$

**Table 1:** Comparison of pCR and non-pCR expression of candidate small RNAs

Variables	pCR (*n* = 30)^a^	Non-pCR (*n* = 69)^a^	*p* Value^b^
*isomiR*			
isomiR of let-7b-5p (3' deletion T and 3' addition CTC)	160.1 (0–832.9)	139.1 (0–518.1)	**0.019**
isomiR of miR-93-5p (3' addition TTTG)	326.7 (0–989.0)	207.4 (0–2035.4)	0.091
*tRNA-derived fragment*			
5'-half derived from tRNA-Gly-CCC/GCC	69.2 (0.0–210.9)	9.1 (0.0–730.0)	0.074
5'-tRF derived from tRNA-Gly-CCC/GCC	57.9 (0.0–1944.6)	44.0 (0.0–730.0)	**0.011**
i-tRF derived from tRNA-Gly-CCC/GCC	182.7 (0.0–756.0)	56.7 (0.0–493.1)	**0.007**
tsRNA group from tRNA-Gly-CCC/GCC	269.5 (26.4–2061.6)	144.8 (0–1180.2)	**< 0.001**

pCR, pathologic complete response; RNA, small ribonucleic acid; isomiR, isoform of micro-RNA; tRNA, transfer RNA; tRF, transfer RNA fragment; i-tRF, internal transfer RNA fragment; tsRNA, transfer RNA-derived small RNA

^a^Continuous variables are expressed as medians (ranges)

^b^Variables in bold font are significant (*p* < 0.05)

### Predictive Model of pCR With Clinical Factors

A comparison of clinical factors between the pCR and non-pCR groups is shown in Table 2. The non-pCR group had a greater incidence of tumor cells in the primary biopsy than the pCR group after NACRT (pCR group [0 %] vs non-pCR group [15.9 %]; *p* = 0.014) and greater CEA values after NACRT (pCR group median [1.7 ng/mL] vs non-pCR group median [2.7 ng/mL]; *p* = 0.004). According to the RECIST criteria, compared with the non-pCR group, the pCR group had higher cCR rates (pCR group [40 %] vs non-pCR group [21.7 %]; *p* = 0.061) and greater %ΔSUVmax of the primary tumor (pCR group median [76.7 %] vs non-pCR group median [69.6 %]; *p* = 0.05). Thus, these variables were identified as candidate predictors. The pCR predictive model for clinical factors was obtained via logistic regression analysis. Figure 2b shows the diagnostic performance of the pCR model (accuracy, precision, recall, F-measure, and AUC).

**Table 2:** Clinical characteristics of the patients with pCR and non-pCR

Variables	pCR (*n* = 30) *n* (%)	Non-pCR (*n* = 69) *n* (%)	*p* Value^a^
Median age: years (range)	64 (35–73)	65 (46–80)	0.218
Male/female	26 (86.7)/4 (13.3)	56 (81.2)/13 (13.8)	0.504
Median tumor diameter: mm (range)	50 (20–100)	50 (30–100)	0.613
cT1-2/cT3-4	4 (13.3)/26 (86.7)	10 (14.5)/59 (85.5)	0.576
cN0/cN1-3	9 (30)/21 (70)	14 (20.3)/55 (79.7)	0.293
cM0/cM1 (LYM)	24 (80)/6 (20)	60 (87.0)/9 (23.0)	0.274
Endoscopic evaluation: cCR/non-CR	15 (50)/15 (50)	25 (36.2)/44 (63.8)	0.199
Overall response: cCR/non-CR	12 (40)/18 (60)	15 (21.7)/54 (78.3)	0.061
Endoscopic biopsy: positive/negative	0 (0)/30 (100)	11 (15.9)/58 (84.1)	**0.014**
*Tumor marker of post-NACRT*			
Median CEA: ng/mL (range)	1.7 (0.7–5.7)	2.7 (0.6–9.8)	**0.004**
Median SCC: ng/mL (range)	1.0 (0.4–2.8)	1.2 (0.5–14.9)	0.251
Median %ΔSUVmax of the primary lesion (range)	76.7 (14.3–95)	69.6 (23.0–89.8)	0.050

pCR, pathologic complete response; cM1(LYM), clinical metastasis to the supraclavicular lymph node; cCR, clinical complete response; NACRT, neoadjuvant chemoradiotherapy; CEA, carcinoembryonic antigen; SCC, squamous cell carcinoma antigen; SUVmax, maximum standardized uptake value; %ΔSUVmax, percentage change in SUVmax before and after NACRT

Continuous variables are expressed as medians (range), whereas qualitative variables are expressed as numbers (%)

^a^Variables in bold font are significant (*p* < 0.05)

The predictive model with clinical factors (CF-PM) was calculated as follows:

$$\begin{aligned} {\text{Score }} = & - 0.0{2913 } + \, 0.{478113 } \times [{\text{cCR}}:{1},{\text{ noncCR}}:0] + \, 0.0{17915 } \\ & \times \,[\% \Delta {\text{SUVmax}}] - \, 0.{51754 } \times [{\text{post}} - {\text{CEA}}] - { 1}.{831}0{5 } \\ & \times \,[{\text{postbiopsy}}\,{\text{positive:1}},{\text{ negative:}}0]. \\ \end{aligned}$$

### Predictive Model for pCR From Clinical Factors and sRNA

Combined predictive models of pCR were established with four clinical factors and one to three candidate sRNAs in the order of increasing odds ratios via cross-validation. Figure 2c depicts the predictive ability of each of the three pCR predictive models. After combining several clinical factors and sRNAs, the predictive model with four clinical factors, two isomiRs, and one tsRNA showed the highest AUC. In addition, this predictive model was superior to that with clinical factors and sRNAs alone in terms of accuracy, precision, reproducibility, and F-measure. The combined predictive model (C-PM) was calculated as follows:

$$\begin{aligned} & - {1}.{2}0{176 } + \, 0.{3}0{144 } \times [{\text{cCR}}:{1},{\text{ noncCR}}:0] + \, 0.0{11833 } \\ & \quad \times [\% \Delta {\text{SUVmax}}] + \, 0.00{3975 } \times [{\text{isomiR of let}} - {\text{7b}} - {\text{5p}}] \\ & \quad + \, 0.00{2576 } \times [{\text{tsRNA group derived from Gly}} - {\text{CCC}}/{\text{GCC}}] \\ & \quad + \, 0.000{149 } \times [{\text{isomiR of miR}} - {93} - {\text{5p}}] - \, 0.{44751 } \\ & \quad \times [{\text{post}} - {\text{CEA}}] - { 2}.{43116 } \\ & \quad \times [{\text{postbiopsy positive}}:{ 1},{\text{ negative}}: \, 0]. \\ \end{aligned}$$

### Association Between the pCR Predictive Model and Patient Prognosis

The distributions of CF-PM, SR-PM, and C-PM in patients with and without pCR are shown in Fig. S1. Based on receiver operating characteristic curve analysis (Fig. 2d), the cutoff values for CF-PM, SR-PM, and C-PM were set at 0.074, − 0.112, and − 0.589, respectively. The C-PM cutoff of − 0.589 had a sensitivity of 0.966 and a specificity of 0.574, and the accuracy of the C-PM was 0.76.

Table 3 shows the results of logistic regression analysis for predicting pCR. Multivariate analysis 1 was performed using CF-PM, SR-PM, and clinical factors excluded from CF-PM. Multivariate analysis 1 demonstrated that the significant factors were CF-PM (odds ratio [OR], 9.18; 95 % confidence interval [CI] 2.80–30.20; *p* < 0.001) and SR-PM (OR, 8.19; 95% CI 2.44–27.47; *p* < 0.001). Multivariate analysis 2 was performed using C-PM and clinical factors other than C-PM, and C-PM was found to be a significant predictor of pCR (OR, 89.41; 95% CI 8.10–987.5; *p* < 0.001).

**Table 3:** Uni- and multivariate analyses for predicting pCR

Variables	Univariate analysis			Multivariate analysis 1			Multivariate analysis 2		
	OR	95 % CI	*p* Value^a^	OR	95 % CI	*p* Value^a^	OR	95 % CI	*p* Value^a^
cT3-4	1.10	0.32–3.84	0.879	3.32	0.64–17.38	0.155	3.16	0.68–14.71	0.142
cN1-3	0.59	0.22–1.58	0.296	0.32	0.09–1.20	0.091	0.37	0.11–1.25	0.109
cM1(LYM)	1.67	0.54–5.19	0.378	4.99	0.997–24.9	0.050	3.26	0.77–13.82	0.108
%ΔSUVmax	1.02	0.99–1.06	0.119						
post-CEA	0.57	0.38–0.87	**0.009**						
cCR (overall response)	2.40	0.950–6.07	0.064						
CF-PM high	8.58	3.05–24.16	**< 0.001**	9.18	2.80–30.20	**< 0.001**			
SR-PM high	7.02	2.62–18.82	**< 0.001**	8.19	2.44–27.47	**< 0.001**			
C-PM high	37.66	484–293.0	**< 0.001**				89.41	8.10–987.5	**< 0.001**

*pCR* pathologic complete response, *OR* odds ratio, *CI* confidence interval, *cM1* (LYM) clinical metastasis to the supraclavicular lymph node, *%ΔSUVmax* percent change in SUVmax before and after NACRT CEA carcinoembryonic antigen, *cCR* clinical complete response, *CF-PM* predictive model with clinical factors, *C-PM* combined predictive model, *SR-PM* predictive model with small RNA

^a^Variables in bold font are significant (*p* < 0.05)

### Relationships Between Prognosis and pCR, Clinical Factors, and sRNA-Based Predictive Models

**Fig. 3:**
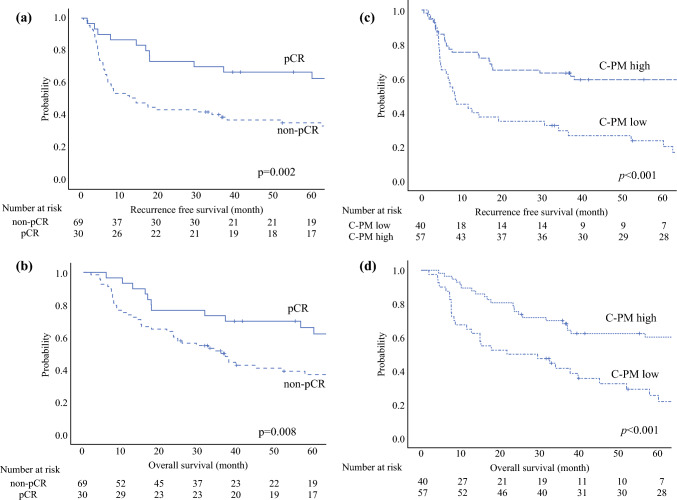
Kaplan–Meier curves of (**a**) recurrence-free survival and (**b**) overall survival for patients with pCR and non-pCR, and Kaplan–Meier curves of (**c**) recurrence-free survival and (**d**) overall survival for patients with the combined predictive model (C-PM) high and low. pCR, pathologic complete response

Table 4 shows the results of Cox regression analysis predicting RFS and OS. Multivariate analysis 1 included pCR as a factor, whereas multivariate analysis 2 used C-PM instead of pCR. The pCR was a predictor of RFS (hazard ratio [HR], 0.36; 95% CI 0.19–0.67;* p* = 0.001) and OS (HR, 0.42; 95% CI 0.22–0.78; *p* = 0.006). Similarly, C-PM was a predictor of prognosis in terms of RFS (HR, 0.43; 95% CI 0.25–0.73;* p* = 0.002) and OS (HR, 0.40; 95% CI 0.23–0.69; *p* = 0.001). In addition, Kaplan–Meier curve analysis showed that C-PM significantly stratified RFS and OS after esophagectomy following NACRT (Fig. 3).

**Table 4:** Uni- and multivariate analyses for recurrence-free survival (RFS) and overall survival (OS)

Variables	Univariate analysis (RFS)			Multivariate analysis 1 (RFS)			Multivariate analysis 2 (RFS)			Univariate analysis (OS)			Multivariate analysis 1 (OS)			Multivariate analysis 2 (OS)		
	HR	95 % CI	*p* Value^a^	HR	95 % CI	*p* Value^a^	HR	95 % CI	*p* Value^a^	HR	95 % CI	*p* Value^a^	HR	95 % CI	*p* Value^a^	HR	95 % CI	*p* Value^a^
Age ≥65 years	1.3	0.8–2.1	0.346	1.2	0.7–2.0	0.474	1.2	0.7–1.9	0.590	1.4	0.8–2.4	0.189	1.4	0.8–2.3	0.255	1.3	0.8–2.3	0.406
Female	0.7	0.3–1.4	0.303	0.6	0.3–1.3	0.239	0.7	0.4–1.5	0.394	0.6	0.3–1.3	0.204	0.6	0.3–1.3	0.168	0.7	0.3–1.4	0.295
ECOG PS 1	1.5	0.6–3.7	0.402	1.6	0.6–4.3	0.371	1.2	0.4–3.4	0.685	2.1	0.8–5.2	0.123	2.4	0.9–6.9	0.095	1.8	0.7–5.2	0.252
cT3–4	1.2	0.6–2.4	0.603	1.3	0.7–2.7	0.431	1.1	0.5–2.2	0.895	1.0	0.5–2.1	0.931	1.0	0.5–2.1	0.949	0.8	0.4–1.7	0.578
cN1–3	1.6	0.8–2.9	0.163	1.4	0.8–2.7	0.271	1.5	0.8–2.9	0.202	1.6	0.8–2.9	0.170	1.7	0.8–3.4	0.143	1.8	0.9–3.5	0.109
cM1 (LYM)	1.4	0.8–2.8	0.270	1.7	0.9–3.3	0.135	1.3	0.6–2.5	0.535	1.4	0.7–2.7	0.345	1.5	0.7–2.9	0.281	1.1	0.5–2.2	0.863
pCR	0.4	0.2–0.7	**0.003**	0.4	0.2–0.7	**0.001**				0.4	0.2–0.8	**0.010**	0.4	0.2–0.7	**0.006**			
C-PM high	0.4	0.2–0.7	**<0.001**				0.4	0.3–0.7	**0.002**	0.4	0.2–0.7	**<0.001**				0.4	0.2–0.7	**0.001**

HR, hazard ratio; CI, confidence interval; ECOG PS; Eastern Cooperative Oncology Group Performance Status; cM1(LYM), clinical metastasis to the supraclavicular lymph node; pCR, pathologic complete response; C-PM, combined predictive model

^a^Variables in bold font are significant (*p* < 0.05)

## Discussion

The findings showed that NACRT results in robust local control and a greater pCR rate than preoperative adjuvant chemotherapy.^4,28^ However, concerns persist regarding the potential effect that late toxicity of NACRT has on survival benefit.^29^ Thus, the toxicity of NACRT and subsequent surgical invasion may affect patient prognosis, highlighting the urgent need for preoperative prediction for patients who achieve a pCR and may not undergo esophagectomy.

Reports on the preoperative prediction of pCR after NAT using sRNA are limited. Skinner et al.^30^ reported that the pCR rate after NACRT differed based on four miRNA expression profiles in esophageal adenocarcinoma tissue. In addition to miRNA expression profiles in tissues, reports show that liquid biopsy is less invasive and predicts therapeutic efficacy as an esophageal cancer diagnostic biomarker. Okuno et al.^24^ reported the usefulness of a diagnostic model containing four miRNAs and three messenger RNAs from blood sample combined with tumor size, tumor location, and lymphatic invasion in predicting the therapeutic response to NAT. To the best of our knowledge, reports on sRNA prediction models of pCR in ESCC patients using post-NACRT blood-derived ncRNA are lacking.

The pCR predictive model showed elevated let-7b and miR-93 isomiRs as predictors, and numerous reports descibe their function and therapeutic sensitivity. Low expression of let-7b and let-7c derived from esophageal cancer tissues before preoperative cisplatin-based chemotherapy is correlated with poor pathologic tissue response by regulating the interleukin-6/STAT3 pathway.^31^ *In vitro* research has shown that decreased let-7b promotes the proliferation and motility of esophageal cancer cell lines via KIAA1377 and that let-7b expression suppresses esophageal cancer cell lines.^32^

Wang et al.^33^ reported that decreased plasma miR-93 expression predicted PR or CR in patients with ESCC undergoing definitive chemoradiotherapy. Our study detected an elevated level of the miR-93 isoform in patients who achieved pCR, contrary to existing reports, possibly because existing reports have predicted its efficacy for predicting clinical response of PR above using RECIST, whereas our study predicted pCR, classifying several patients who achieved PR using RECIST as having non-pCR. Moreover, elevated miR-93 levels increase sensitivity to radiotherapy^34^ and chemotherapy^35^ in breast cancer. Reports on the association between sensitivity to NAT and miR-93 in ESCC are limited and should be explored further.

Recent developments in analytical methods, such as NGS, have led to increased interest in the association between tRFs and tumors.^36,37^ However, only limited reports are available. Huang et al.^38^ recently reported a blood biomarker for esophageal cancer using tsRNAs. This is the first report on the use of tsRNA in addition to isomiR to predict pCR after NAT, and the ability of blood ncRNAs is significant in predicting the disappearance of primary tumors and metastatic lymph nodes.

The usefulness of active surveillance for patients who respond well to NACRT is currently under investigation. The preSANO trial, which examined the ability to detect tumor remnants after NACRT, reported that for patients with ≥ 11 % tumor remnants (TGR 3–4), conventional biopsy techniques yielded false negatives for 28 % of patients. Furthermore, accurate determination of the N stage is challenging.^39^ One strength of this study was the establishment of a model to identify patients with pT0M0M0 disease who are eligible for active surveillance because even if the primary tumor had disappeared (grade 3), it was considered non-pCR when accompanied by lymph node metastasis. Similar to our current results, we previously reported that the rate of change in the SUVmax on PET-CT is a preoperative predictor of NACRT efficacy.^40^ In addition, higher CEA values after NACRT were a risk factor for non-pCR in this study.

Among tumor markers, CEA, SCC-Ag, and carbohydrate antigen 19-9 (CA-19-9) are reported to be useful diagnostic and prognostic markers for ESCC. Yang et al.^41^ reported that among these tumor markers, CEA and CA-19-9 were predictors of OS. Similarly, high CEA levels were reported to be a poor prognostic factor in a nationwide multicenter retrospective study by the Japanese Esophageal Society.^42^ To our knowledge, there are no reports on the usefulness of CEA in predicting pCR, but pCR is considered a prognostic surrogate maker, and a high CEA level may indicate a high probability of tumor remnant.

In the current study, positive preoperative biopsy was included as a factor in the prediction model. However, because false-positive biopsies have been reported,^43,44^ a positive biopsy after NACRT may not definitely guarantee a non-CR, but may only aid diagnosis. Moreover, comprehensive pCR prediction in combination with other factors is considered significant. In addition, SR-PM, CF-PM, and C-PM all were significantly higher in the pCR group, even after the exclusion of patients with a positive preoperative biopsy (Fig. S2).

The pCR predictive model comprising ncRNA was a significant predictor of pCR, and the addition of clinical factors improved its predictive ability, suggesting the possibility of predicting pCR before surgery. Similarly, OS and RFS were stratified using the obtained predictive model, which compared the difference between patients with and without pCR, indicating C-PM as a surrogate marker for prognosis after esophagectomy following NACRT.

This study had a few limitations. First, it was a single-center retrospective study with a relatively small sample.

Second, blood samples before NACRT treatment were unavailable, and changes in blood sRNA levels before and after treatment could not be verified. Additionally, several reports have described changes in blood miRNAs after irradiation,^45–47^ and the possibility that the results of this study also were influenced by NACRT itself cannot be denied. However, this could not be verified due to sample limitations. Nevertheless, this study compared contemporaneous serum samples from patients with and without pCR who received equivalent NACRT, and the effect of NACRT itself is expected to be minimal.

Third, tumor tissue was unavailable for pCR cases, making it impossible to verify whether the sRNA in the blood was derived from the tumor or the surrounding tumor environment due to patient factors. However, the ability of blood ncRNAs, which reflect the tumor environment, to predict the disappearance of all tumors is significant. The sRNA changes in tumor tissue and blood with NAT are of great interest and need to be prospectively validated at multiple centers in the future. In addition, because pCR is a pathologic diagnostic concept, some pCR cases recurred. However, the recurrence rate was significantly lower in the pCR group than in the non-pCR group, and preoperative prediction of pCR as a strong prognostic marker is useful in terms of broadening the range of treatment options.

In conclusion, sRNA obtained from blood samples after NACRT is useful for the preoperative prediction of pCR. Furthermore, the addition of clinical factors to the sRNA predictive model improved its predictive ability. Preoperative prediction of pCR may be useful for patient selection in future organ-preserving approaches.

## Supplementary Information

Below is the link to the electronic supplementary material.

Supplementary file1 (DOCX 239 KB)

Supplementary file2 (XLSB 79 KB)

Supplementary file3 (XLSB 7011 KB)

Supplementary file4 (XLSB 3241 KB)

## Data Availability

Next-generation sequencing data generated in this study are within its supporting information files. Other data supporting the findings are available from the corresponding author upon request.
