# Supplementary material for: Prediction of Pathologic Complete Response in Esophageal Squamous Cell Carcinoma Using Preoperative Serum Small Ribonucleic Acid Obtained After Neoadjuvant Chemoradiotherapy
Source: Ann Surg Oncol. 2024 Oct 17;32(1):570–80. doi: 10.1245/s10434-024-16247-z (PMC11659344; doi:10.1245/s10434-024-16247-z)

**Supplementary Materials - Index**

[Table S1. Clinical and pathological characteristics of all patients 2](#_Toc173587844)

[Figure_S1. Distribution of (a) predictive model with clinical factors (CF-PM), (b) SR-predictive model with small RNA (SR-PM), (c) combined predictive model (C-PM) in pCR and non-pCR. .3](#_Toc173587845)

[Figure_S2. Distribution of (a) predictive model with clinical factors (CF-PM), (b) SR-predictive model with small RNA (SR-PM), (c) combined predictive model (C-PM) in pCR and non-pCR with negative biopsy after NACRT. 4](#_Toc173587846)

**Supplementary Figures and Tables**

# Table S1. Clinical and pathological characteristics of all patients

| Variables | n=99 (%) |
| --- | --- |
| Age (years) (median, range) | 64 (35–80) |
| Men/Women | 82 (82.8%)/17 (17.2%) |
| Tumor diameter (mm) (median, range) | 50 (20–100) |
| TNM stage |  |
| cT1/cT2/cT3/cT4 | 2 (2.0%)/12 (12.1%)/84 (84.8%)/1 (1.0%) |
| cN0/cN1/cN2/cN3 | 23 (23.2%)/49 (49.5%)/27 (27.2%)/0(0%) |
| cM0/cM1 (LYM) | 84 (84.8%)/15 (15.2%) |
| cStage 1/2/3/4 | 0 (0%)/26 (26.3%)/57 (57.6%)/16 (16.2%) |
| Treatment response |  |
| cCR (endoscopic evaluation after NACRT) | 40 (40.4%) |
| cCR (overall response after NACRT) | 27 (27.3%) |
| Endoscopic biopsy after NACRT: positive | 11 (11.1%) |
| Tumor marker of post-NACRT |  |
| CEA | 1.6 (0.8–2.7) |
| SCC | 0.9 (0.7–2.8) |
| SUVmax of the primary lesion |  |
| %ΔSUVmax | 73.0 (14.3–95.0) |
| Pathological diagnosis |  |
| pT0/pT1/pT2/pT3/pT4 | 41 (41.4%)/11 (11.1%)/20 (20.2%)/26 (26.3%)/1 (1.0%) |
| pN0/pN1/pN2/pN3 | 55 (55.6%)/33 (33.3%)/8 (8.1%)/3 (3.0%) |
| pM0/pM1(LYM) | 95 (96.0%)/4 (4.0%) |
| Pathological treatment response |  |
| Grade 3 (pCR, pN0, and pM0) | 30 (30.3%) |
| Grade 3 (pT0, pN1-2, and pM0) | 11 (11.1%) |
| Grade 2/Grade 1 | 39 (39.4%)/19 (19.2%) |

Continuous variables are expressed as medians (range), whereas qualitative variables are expressed as numbers (%).

**Abbreviations:** pCR, pathological complete response; cM1(LYM), clinical metastasis to the supraclavicular lymph node; NACRT, neoadjuvant chemoradiotherapy; cCR, clinical complete response; CEA, carcinoembryonic antigen; SCC, squamous cell carcinoma antigen; SUVmax, maximum standardized uptake value; %ΔSUVmax, percent change in SUVmax before and after NACRT

Figure_S1. Distribution of (a) predictive model with clinical factors (CF-PM), (b) SR-predictive model with small RNA (SR-PM), (c) combined predictive model (C-PM) in pCR and non-pCR.


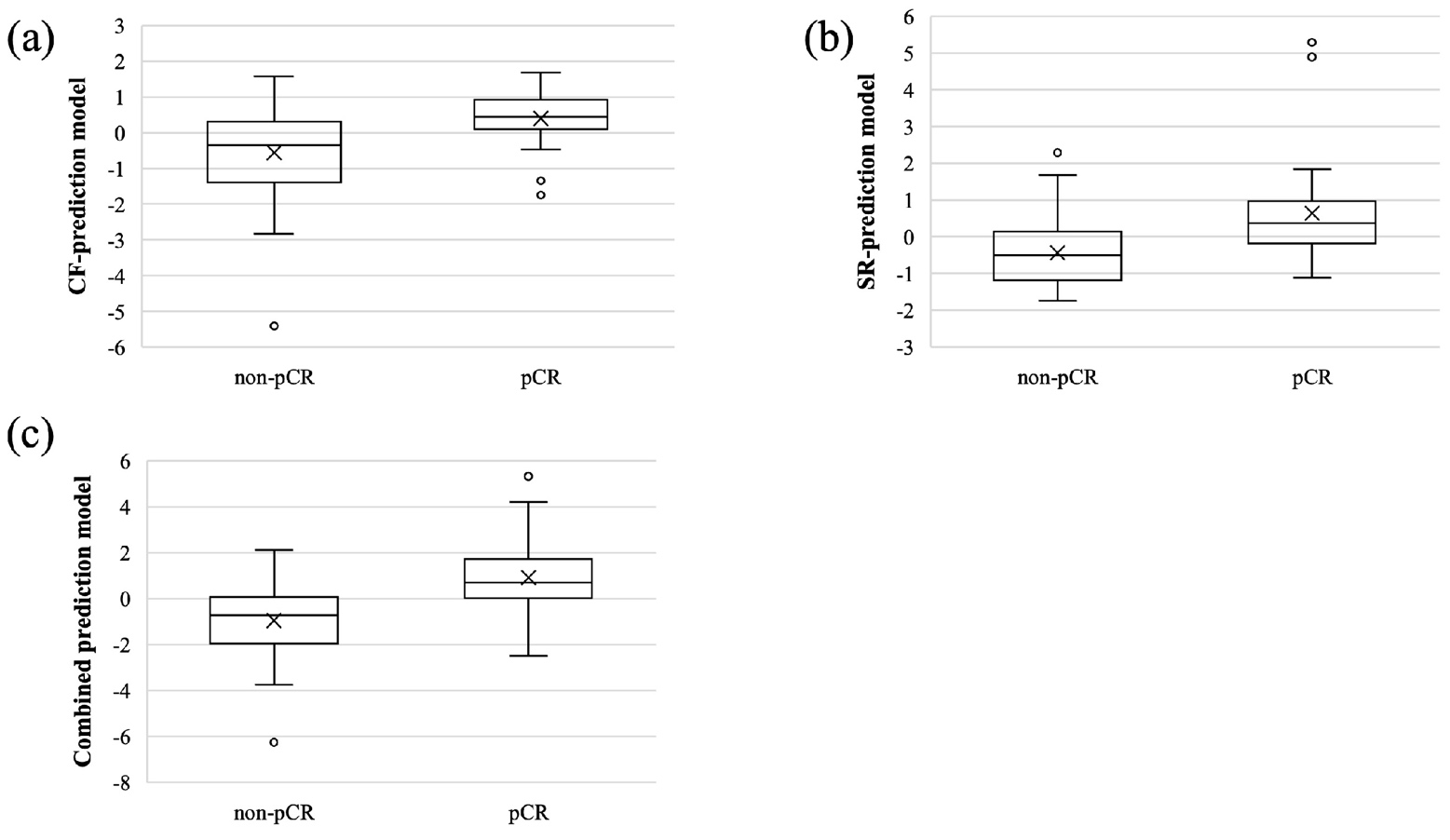


Figure_S2. Distribution of (a) predictive model with clinical factors (CF-PM), (b) SR-predictive model with small RNA (SR-PM), (c) combined predictive model (C-PM) in pCR and non-pCR with negative biopsy after NACRT.


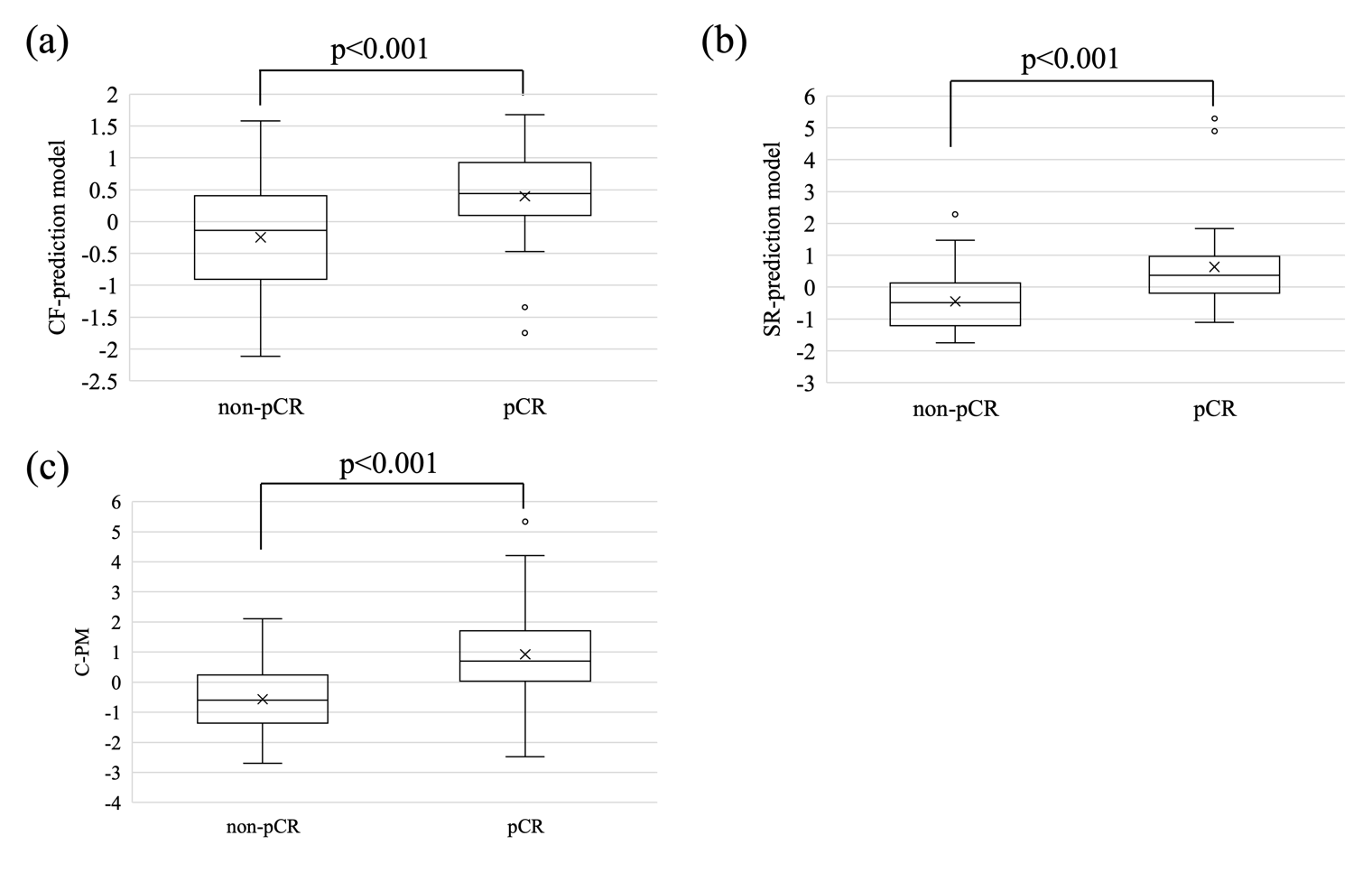

Supplement: Supplementary file 1 — Supplementary file1 (DOCX 239 KB) [file 10434_2024_16247_MOESM1_ESM.docx]
